# Supplementary material for: Multi‐dataset identification of innovative feature genes and molecular mechanisms in keratoconus
Source: J Cell Mol Med. 2024 Sep 19;28(18):e70079. doi: 10.1111/jcmm.70079 (PMC11412914; doi:10.1111/jcmm.70079)
Supplement: Supplementary file 2 — Material S2. [file JCMM-28-e70079-s002.zip › 7.GSEA analysis/4.MYRF/KEGG_T_CELL_RECEPTOR_SIGNALING_PATHWAY.html]

Details for gene set KEGG\_T\_CELL\_RECEPTOR\_SIGNALING\_PATHWAY[GSEA]

|  || Dataset | MYRF.group.cls#h\_versus\_l |
| Phenotype | group.cls#h\_versus\_l |
| Upregulated in class | l |
| GeneSet | KEGG\_T\_CELL\_RECEPTOR\_SIGNALING\_PATHWAY |
| Enrichment Score (ES) | -0.37349796 |
| Normalized Enrichment Score (NES) | -1.3746729 |
| Nominal p-value | 0.07692308 |
| FDR q-value | 0.2525429 |
| FWER p-Value | 0.903 |
Table: GSEA Results Summary

  

Fig 1: Enrichment plot: KEGG\_T\_CELL\_RECEPTOR\_SIGNALING\_PATHWAY      
 Profile of the Running ES Score & Positions of GeneSet Members on the Rank Ordered List

  

| SYMBOL | TITLE | RANK IN GENE LIST | RANK METRIC SCORE | RUNNING ES | CORE ENRICHMENT || 1 | PIK3R5 | na | 661 | 0.528 | 0.0124 | No |
| 2 | NFATC2 | na | 1281 | 0.423 | 0.0196 | No |
| 3 | PPP3R2 | na | 2013 | 0.356 | 0.0197 | No |
| 4 | GRAP2 | na | 2192 | 0.342 | 0.0349 | No |
| 5 | CHP2 | na | 3253 | 0.283 | 0.0211 | No |
| 6 | CBLB | na | 3959 | 0.256 | 0.0159 | No |
| 7 | ZAP70 | na | 5428 | 0.210 | -0.0140 | No |
| 8 | ITK | na | 6002 | 0.192 | -0.0191 | No |
| 9 | CD247 | na | 6238 | 0.185 | -0.0149 | No |
| 10 | SOS1 | na | 6706 | 0.172 | -0.0181 | No |
| 11 | PIK3R1 | na | 7105 | 0.161 | -0.0200 | No |
| 12 | MAPK9 | na | 8409 | 0.129 | -0.0500 | No |
| 13 | MAPK12 | na | 8572 | 0.125 | -0.0472 | No |
| 14 | NCK2 | na | 8807 | 0.120 | -0.0468 | No |
| 15 | KRAS | na | 9371 | 0.108 | -0.0567 | No |
| 16 | PIK3CG | na | 9794 | 0.100 | -0.0629 | No |
| 17 | CBL | na | 9933 | 0.097 | -0.0611 | No |
| 18 | CD4 | na | 10052 | 0.095 | -0.0589 | No |
| 19 | AKT2 | na | 10210 | 0.092 | -0.0579 | No |
| 20 | MAPK11 | na | 10606 | 0.085 | -0.0643 | No |
| 21 | MAPK13 | na | 10951 | 0.079 | -0.0696 | No |
| 22 | PDPK1 | na | 11113 | 0.076 | -0.0697 | No |
| 23 | PIK3CA | na | 11255 | 0.074 | -0.0693 | No |
| 24 | DLG1 | na | 11275 | 0.074 | -0.0655 | No |
| 25 | CD3D | na | 11300 | 0.073 | -0.0618 | No |
| 26 | VAV2 | na | 11454 | 0.071 | -0.0620 | No |
| 27 | VAV3 | na | 11728 | 0.067 | -0.0659 | No |
| 28 | ICOS | na | 11751 | 0.067 | -0.0625 | No |
| 29 | MAPK14 | na | 11965 | 0.064 | -0.0649 | No |
| 30 | CD3G | na | 12252 | 0.060 | -0.0696 | No |
| 31 | CD28 | na | 12283 | 0.059 | -0.0669 | No |
| 32 | PIK3R2 | na | 12535 | 0.056 | -0.0708 | No |
| 33 | SOS2 | na | 12783 | 0.053 | -0.0748 | No |
| 34 | PLCG1 | na | 12816 | 0.052 | -0.0726 | No |
| 35 | IKBKB | na | 12994 | 0.050 | -0.0748 | No |
| 36 | PDCD1 | na | 13374 | 0.045 | -0.0830 | No |
| 37 | PRKCQ | na | 13573 | 0.043 | -0.0862 | No |
| 38 | MAP2K2 | na | 14012 | 0.038 | -0.0966 | No |
| 39 | CHUK | na | 14348 | 0.034 | -0.1042 | No |
| 40 | MAPK3 | na | 14382 | 0.034 | -0.1032 | No |
| 41 | CBLC | na | 14386 | 0.034 | -0.1013 | No |
| 42 | CD3E | na | 15808 | 0.020 | -0.1411 | No |
| 43 | JUN | na | 16671 | 0.011 | -0.1654 | No |
| 44 | PIK3CB | na | 17199 | 0.007 | -0.1802 | No |
| 45 | PPP3R1 | na | 17799 | 0.001 | -0.1974 | No |
| 46 | PAK4 | na | 17893 | 0.001 | -0.2001 | No |
| 47 | AKT1 | na | 18101 | -0.001 | -0.2059 | No |
| 48 | RHOA | na | 18827 | -0.008 | -0.2264 | No |
| 49 | CHP1 | na | 19165 | -0.012 | -0.2354 | No |
| 50 | PAK3 | na | 19225 | -0.013 | -0.2363 | No |
| 51 | MAP2K7 | na | 19405 | -0.015 | -0.2406 | No |
| 52 | GSK3B | na | 19466 | -0.016 | -0.2414 | No |
| 53 | MAPK1 | na | 20406 | -0.027 | -0.2669 | No |
| 54 | PIK3CD | na | 20433 | -0.027 | -0.2661 | No |
| 55 | PIK3R3 | na | 20464 | -0.027 | -0.2653 | No |
| 56 | FOS | na | 20589 | -0.029 | -0.2672 | No |
| 57 | NFATC3 | na | 21139 | -0.035 | -0.2810 | No |
| 58 | IKBKG | na | 21687 | -0.042 | -0.2943 | No |
| 59 | RAF1 | na | 21720 | -0.042 | -0.2927 | No |
| 60 | TEC | na | 21745 | -0.043 | -0.2909 | No |
| 61 | PAK6 | na | 21811 | -0.043 | -0.2902 | No |
| 62 | HRAS | na | 21856 | -0.044 | -0.2888 | No |
| 63 | PPP3CA | na | 22219 | -0.049 | -0.2964 | No |
| 64 | CARD11 | na | 22759 | -0.056 | -0.3086 | No |
| 65 | PPP3CB | na | 22763 | -0.056 | -0.3054 | No |
| 66 | CD8B | na | 23062 | -0.061 | -0.3104 | No |
| 67 | CDC42 | na | 23297 | -0.064 | -0.3133 | No |
| 68 | CTLA4 | na | 23769 | -0.072 | -0.3227 | No |
| 69 | GRB2 | na | 24215 | -0.079 | -0.3308 | No |
| 70 | PAK2 | na | 24412 | -0.082 | -0.3315 | No |
| 71 | LAT | na | 24444 | -0.083 | -0.3275 | No |
| 72 | CDK4 | na | 24674 | -0.088 | -0.3289 | No |
| 73 | MAP3K7 | na | 24724 | -0.088 | -0.3251 | No |
| 74 | PTPRC | na | 24862 | -0.091 | -0.3236 | No |
| 75 | NCK1 | na | 25117 | -0.095 | -0.3253 | No |
| 76 | CD8A | na | 25192 | -0.097 | -0.3217 | No |
| 77 | NFKBIB | na | 25462 | -0.102 | -0.3234 | No |
| 78 | PTPN6 | na | 25517 | -0.103 | -0.3188 | No |
| 79 | IL5 | na | 26211 | -0.117 | -0.3318 | No |
| 80 | PAK1 | na | 26325 | -0.120 | -0.3280 | No |
| 81 | MALT1 | na | 27090 | -0.137 | -0.3419 | No |
| 82 | NFAT5 | na | 27258 | -0.141 | -0.3383 | No |
| 83 | NFKB1 | na | 28241 | -0.168 | -0.3567 | No |
| 84 | PPP3CC | na | 28351 | -0.171 | -0.3496 | No |
| 85 | VAV1 | na | 28682 | -0.181 | -0.3484 | No |
| 86 | IL10 | na | 28824 | -0.186 | -0.3414 | No |
| 87 | LCK | na | 29935 | -0.221 | -0.3603 | Yes |
| 88 | NRAS | na | 30019 | -0.224 | -0.3494 | Yes |
| 89 | MAP2K1 | na | 30150 | -0.229 | -0.3395 | Yes |
| 90 | CD40LG | na | 30708 | -0.251 | -0.3407 | Yes |
| 91 | RELA | na | 31135 | -0.271 | -0.3369 | Yes |
| 92 | NFKBIA | na | 31525 | -0.292 | -0.3308 | Yes |
| 93 | NFATC1 | na | 31545 | -0.293 | -0.3139 | Yes |
| 94 | RASGRP1 | na | 31683 | -0.300 | -0.3000 | Yes |
| 95 | MAP3K14 | na | 32615 | -0.359 | -0.3056 | Yes |
| 96 | NFATC4 | na | 32887 | -0.379 | -0.2908 | Yes |
| 97 | LCP2 | na | 32895 | -0.380 | -0.2684 | Yes |
| 98 | NFKBIE | na | 32930 | -0.382 | -0.2467 | Yes |
| 99 | IL4 | na | 33045 | -0.391 | -0.2267 | Yes |
| 100 | IFNG | na | 33150 | -0.402 | -0.2058 | Yes |
| 101 | IL2 | na | 33402 | -0.431 | -0.1874 | Yes |
| 102 | BCL10 | na | 33569 | -0.452 | -0.1653 | Yes |
| 103 | FYN | na | 33971 | -0.517 | -0.1461 | Yes |
| 104 | TNF | na | 34328 | -0.609 | -0.1202 | Yes |
| 105 | MAP3K8 | na | 34409 | -0.639 | -0.0845 | Yes |
| 106 | CSF2 | na | 34421 | -0.642 | -0.0466 | Yes |
| 107 | AKT3 | na | 34696 | -0.932 | 0.0009 | Yes |
Table: GSEA details [plain text format]

  

Fig 2: KEGG\_T\_CELL\_RECEPTOR\_SIGNALING\_PATHWAY      
 Blue-Pink O' Gram in the Space of the Analyzed GeneSet

  

Fig 3: KEGG\_T\_CELL\_RECEPTOR\_SIGNALING\_PATHWAY: Random ES distribution      
 Gene set null distribution of ES for **KEGG\_T\_CELL\_RECEPTOR\_SIGNALING\_PATHWAY**
